# Evolutionary mismatch

**DOI:** 10.1093/emph/eoy023

**Published:** 2018-08-08

**Authors:** Melissa B Manus

**Affiliations:** 1 Department of Evolutionary Anthropology, Duke University, Durham, NC, USA; 2 Triangle Center for Evolutionary Medicine Duke University, Durham, NC, USA

## DEFINITION AND BACKGROUND

**Figure 1.**
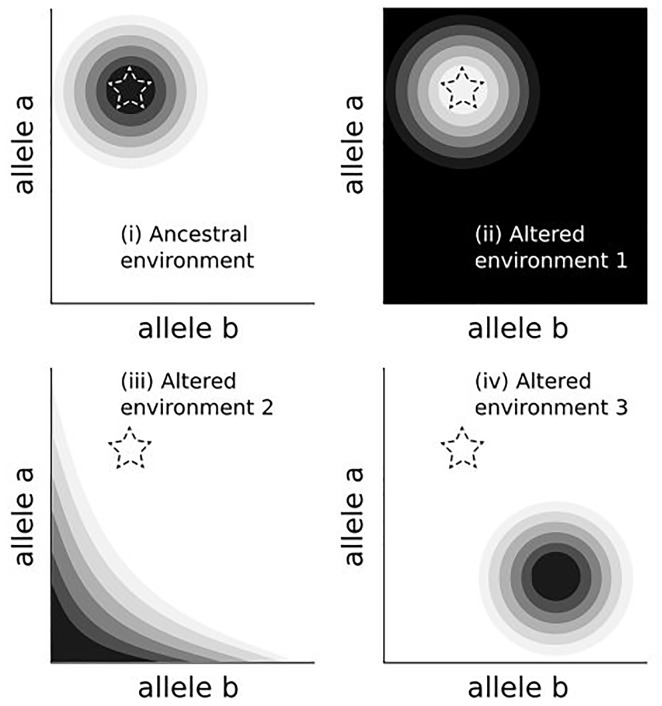
The ancestral environment (i) favours a certain combination of alleles (darker colours). Transitions to modernity produce shifts in the allelic combination that is best suited to the new environment (ii-iv). Mismatch occurs when ancestral alleles (star) persist in new settings where the environment, but not yet genetics, has changed.

The adaptive landscape of the ancestral human environments selected for a suite of genetic, behavioural and physiological traits, many of which persist in contemporary human populations. The transition to modernity [1] rapidly reshaped these environments, yet the slower rate of biological evolution limits phenotypic change. This results in evolutionary mismatch, defined here as the phenomenon by which previously adaptive alleles are no longer favoured in a new environment (Fig. 1). This definition operates across space and time, while other uses of mismatch are applied over the life course [2, 3].

## EXAMPLE IN HUMAN BIOLOGY AND PUBLIC HEALTH

Due to variation in food availability and quality, the ancestral environment favoured efficient energy extraction and storage [4, 5]. These once-adaptive traits become mismatched to industrialized settings with an overabundance of easily accessible, calorie-dense foods. This surplus, coupled with the relatively low energy output required by the modern lifestyle [5], promotes an energy imbalance that contributes to weight gain. Today, obesity is rampant across populations in developed countries and rapidly increasing in developing nations transitioning to the industrialized lifestyle [6].

## EXAMPLE IN CLINICAL MEDICINE

Humans evolved in environments rich with biodiversity, including helminthic worms that co-evolved to regulate our immune systems [7]. Modern medicine and hygiene largely eradicated these helminths from industrialized settings, and their absence is now implicated in a suite of hyper-inflammatory conditions, including multiple sclerosis and irritable bowel disease [7, 8]. Helminthic therapy, which reunites the human body with part of its ancestral biota, has been suggested as a treatment for the immune system’s inappropriate response to a mismatched environment.
